# The role of routine first-trimester ultrasound screening for central nervous system abnormalities: a longitudinal single-center study using an unselected cohort with 3-year experience

**DOI:** 10.1186/s12884-023-05644-z

**Published:** 2023-05-03

**Authors:** Yu Hu, Lijuan Sun, Li Feng, Jingjing Wang, Yantong Zhu, Qingqing Wu

**Affiliations:** 1 Beijing Obstetrics and Gynecology Hospital, Capital Medical University, Beijing, P. R. China; 2 Beijing Maternal and Child Health Care Hospital, Beijing, P. R. China; 3 Department Ultrasound, Beijing Obstetrics and Gynecology Hospital, Capital Medical University, No.251 Yaojiayuan Road, Beijing, Chaoyang District 100026 P. R. China

**Keywords:** First trimester, Ultrasound, Central nervous system abnormalities, Prenatal diagnosis, Early screening

## Abstract

**Background:**

To evaluate the role of a standardized first-trimester scan in screening different kinds of central nervous system malformations and to report a 3-year experience from a tertiary center using an unselected cohort.

**Methods:**

This was a retrospective analysis of prospectively collected data from a single center evaluating first-trimester scans with predesigned standardized protocols performed between 1 May 2017 and 1 May 2020, involving 39,526 pregnancies. All pregnant women underwent a series of prenatal ultrasound scans at 11–14, 20–24, 28–34 and 34–38 weeks of gestation. Abnormalities were confirmed by magnetic resonance imaging, postmortem examination or trained ultrasound professionals. Pregnancy outcomes and some postnatal follow-up were obtained from maternity medical records and telephone calls.

**Results:**

A total of 38,586 pregnancies included in the study. The detection rates of CNS anomalies by ultrasound in the first, second, third and late third trimester were 32%, 22%, 25%, and 16%, respectively. And there were 5% of CNS anomalies missed by prenatal ultrasound. In the first-trimester scan, we diagnosed all cases of exencephaly, anencephaly, alobar holoprosencephaly and meningoencephalocele, and some cases of posterior cranial fossa anomalies (20%), open spina bifida (67%), semilobar holoprosencephaly (75%) and severe ventriculomegaly (8%). Vein of Galen aneurysmal malformation, closed spina bifida, lobar holoprosencephaly, intracranial infection, arachnoid cyst, agenesis of the corpus callosum, cysts of the septum pellucidum and isolated absence of the septum pellucidum were never detected during the first trimester. The abortion rates of fetal CNS anomalies detected by first-trimester scan, second-trimester scan, and third- trimester scan were 96%, 84% and 14%, respectively.

**Conclusions:**

The study showed that almost 1/3 of central nervous system anomalies were detected by the standard first-trimester scan and these cases were associated with a high rate of abortion. Early screening for fetal abnormalities gives parents more time for medical advice and safer abortion if needed. It is therefore recommended that some major CNS anomalies should be screened in the first trimester. The standardized anatomical protocol, consisting of four fetal brain planes, were recommended for routine first trimester ultrasound screening.

## Background

First-trimester ultrasound evaluation has been performed for almost 20 years since 1990s [1–3]. Fetal nuchal translucency (NT), an important indicator of first-trimester scan, could be used to screen for fetal aneuploidy and structural malformations [4, 5]. However, it has been discovered that the increased NT thickness was found to be closely associated with congenital heart diseases [6, 7], rather than central nervous system (CNS) abnormalities. It is therefore insensitive to the prenatal diagnosis of CNS abnormalities using NT thickness alone. With the development of ultrasound technology, the assessment of fetal anatomy has become feasible [8]. In recent years, many studies have shown that the change of intracranial structures such as nasal bone, brain stem (BS), brain stem-to-occipital bone (BSOB), intracranial translucency (IT), cisterna magna (CM) can be observed in fetuses with nervous system abnormalities in early pregnancy [9–11], what’s more, it’s also reported that the position of the choroid plexus of the fourth ventricle was an approach to detected the cystic posterior fossa anomalies [12], low torcular Herophili position and large brainstem-tentorium angle is useful in the diagnosis of Chiari-II malformation [13], and so on. Standardized ultrasound scans have long been used for routine anatomical screening in the second trimester. It is necessary to standardize the scan for central nervous system abnormalities during the first trimester.

The ability of first-trimester ultrasound to detect various malformations has been reported and has been variable [14–18]. However, there are few reports focusing on the ability of first-trimester ultrasound to detect detailed categories of central nervous system anomalies [19]. The aim of this study was to evaluate the role of a standardized first-trimester scan in screening for different kinds of central nervous system malformations and to report a 3-year experience from a tertiary center using an unselected cohort.

## Methods

This was a retrospective analysis of prospectively collected data from a single center evaluating first-trimester scans performed between 1 May 2017 and 1 May 2020, using predesigned standardized protocols. All pregnant women underwent a series of prenatal ultrasound scans at 11–14, 20–24, 28–34 and 34–38 weeks of gestation. All sonographers who performed first-trimester ultrasound scans had to undergo a rigorous training and pass a qualification examination. High-resolution ultrasound devices for fetal anatomy at 11 to 13^+ 6^ weeks’ gestation were used, including GE Voluson E8 and E10, Samsung WS80A, and Philips EPIQ7.

### First-trimester scanning

**Fig. 1:**
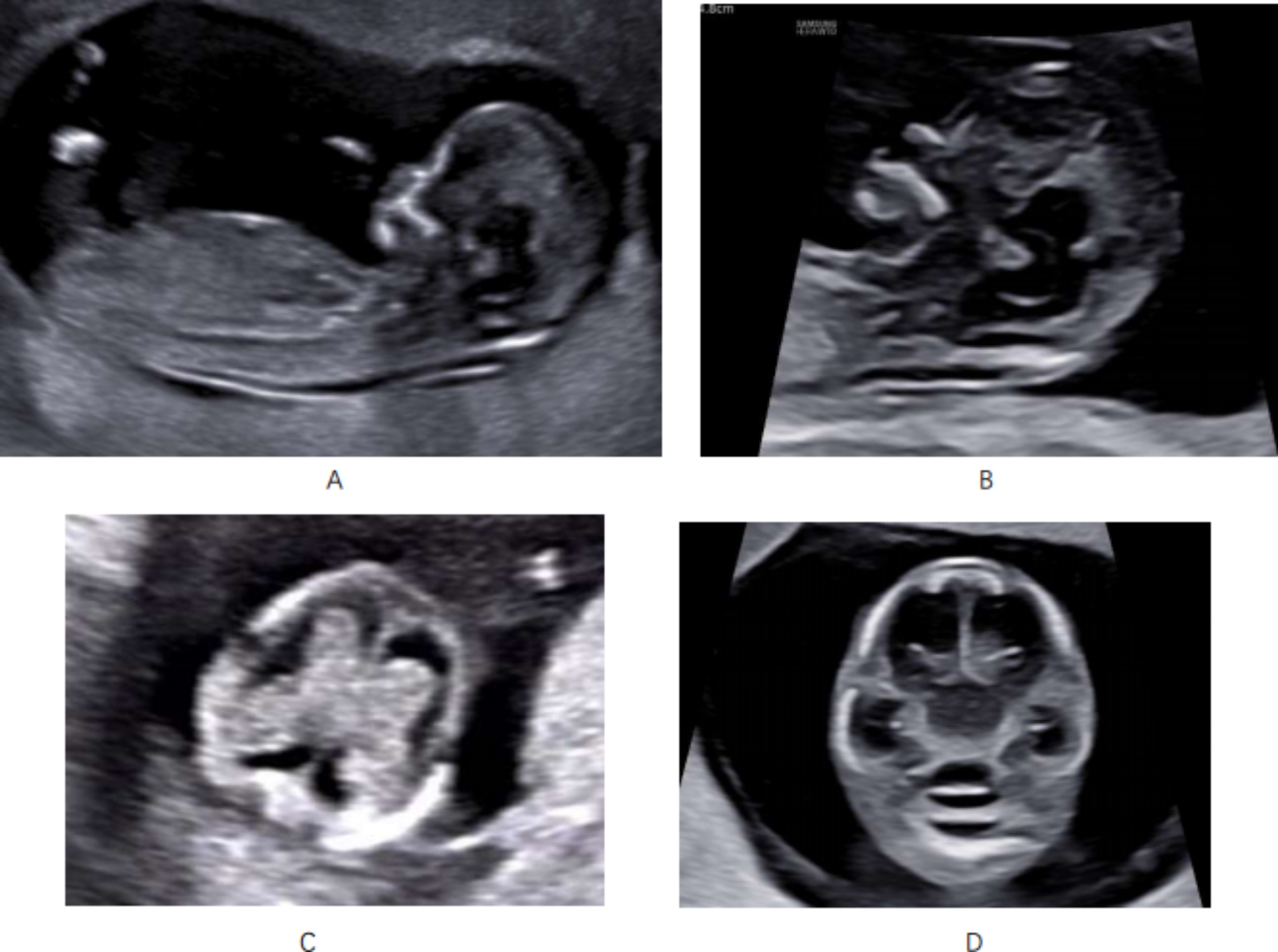
(**A**): midsagittal plane of the fetus; (**B**): midsagittal view of fetal brain (**C**): the transventricular plane (**D**): the transthalamus plane

The four standard views focused on CNS were required for first-trimester ultrasound screening and were as follows (Fig. 1): midsagittal plane of the fetus (Fig. 1, A), midsagittal view of the fetal brain (Fig. 1, B), the transventricular plane (Fig. 1, C), and the transthalamus plane (Fig. 1, D). The Crown-Rump Length (CRL) was measured from the midsagittal plane of the fetus (Fig. 1, A). NT thickness, nasal bone, brain stem, intracranial translucency, cisterna magna and facial outline were observed from the midsagittal view of fetal brain (Fig. 1, B). The choroid plexuses, lateral ventricular and falx cerebri were obtained from the transventricular plane (Fig. 1, C). Thalamus and the integrity of skull were observed from the transthalamus plane (Fig. 1, D).

If fetal improperly position or other reasons impeded the examination, the ultrasound scan should be repeated 30 min later. Transabdominal ultrasonography was always performed. If necessary, transvaginal examination was done with consent of the patients. All fetal anomalies detected during early pregnancy must be confirmed by at least a senior doctor, and usually a repeated examination was suggested after two weeks to make sure the anomalies and possible other abnormalities. All acquired ultrasound images were stored in medical image database. Imaging quality control was performed every month by a senior physician.

### Second- and third-trimester scans and follow-up

When a CNS malformation was detected or suspected at 11–14 weeks, a follow up confirmation scan at 16–18 weeks would be performed in the absence of abortion. If the pregnancy continued, a detailed anatomical examination was performed at 20 to 24 weeks’ gestation following the ISUOG guidelines for routine mid-trimester fetal ultrasound [20], the 28 to 34 weeks and 34 to 38 weeks growth scan were performed sequentially. The fetuses suspected of having central nervous system anomalies would undergo magnetic resonance imaging (MRI) examination at gestational age > 20 weeks. And invasive genetic material testing was usually recommended. Abnormalities were confirmed by postmortem examination or MRI results in cases of pregnancy termination or miscarriage. If neither autopsy nor MRI results were available, abnormalities were confirmed by trained ultrasound professionals. Pregnancy outcomes and a part of postnatal follow-up were obtained from maternity medical records and telephone calls.

### Inclusion and exclusion criteria

Women with a live fetus or twins or multiple pregnancies were eligible for this study if they initiated antenatal care before 14 weeks’ gestation at Beijing Obstetrics and Gynecology hospital, Capital Medical University. All pregnant women were required to undergo a series of prenatal ultrasound scans at 11–14, 20–24, 28–34, and 34–38 weeks’ gestation. Incomplete pregnancy information and ultrasound examinations, miscarriages and fetal death before the first-trimester examination were not included in this study.

### Statistical analysis

Data were analyzed with SPSS version 23.0 software (IBM Corporation, Armonk, NY). Continuous variables were expressed as mean ± SD (standard deviation). Continuous variables between 2 groups were compared by the independent-samples t test, with statistical significance defined as P < 0.05. The first-trimester detection rate and the termination of pregnancy (TOP) rate were expressed as a percentage. The first-trimester detection rate was calculated as the number of fetuses with CNS abnormalities diagnosed or suspected by first-trimester ultrasound divided by the total number of fetuses with CNS abnormalities. The TOP rate was calculated as the number of fetuses with CNS abnormalities diagnosed or suspected in each trimester and terminated finally divided by the total number of fetuses with CNS abnormalities detected in that trimester.

## Results

A total of 39,526 women who were enrolled in Beijing Obstetrics and Gynecology Hospital, Capital Medical University underwent first-trimester screening for abnormalities. There were 940 cases of loss to follow up, unexplained fetal death or miscarriage. The remaining 38,586 pregnancies were included in this study, including 37,169 singleton pregnancies, 1,384 twin pregnancies, and 33 triplet pregnancies. Baseline characteristics of the 141 cases with CNS abnormalities and 38,445 pregnancies with normal CNS were shown in Table 1. There was a statistically significant difference in NT thickness between the abnormal CNS cases group and the normal CNS group in this study.

**Table 1:** Baseline characteristics of the 38,586 pregnancies and 141 cases with CNS abnormalities

Characteristic		Fetuses with normal CNS	Cases with CNS abnormalities	P
Maternal age, years	Range	17–51	24–44	0.242
	Average (SD)	31.5 (4.0)	31.9 (3.9)	
Maternal height, cm	Range	140–186	150–178	0.918
	Average (SD)	162.8(5.0)	162.8(5.3)	
Maternal weight, kg	Range	35.0-99.5	43.5–97.0	0.498
	Average (SD)	59.0 (9.3)	58.4 (9.9)	
Body-mass index, kg/m^2^	Range	13.0–40.0	16.9–37.5	0.443
	Average (SD)	22.2 (3.3)	22.0 (3.5)	
Fetal CRL, mm		63.8 (7.2)	61.3 (8.4)	< 0.001
Fetal NT, mm		1.4 (0.5)	1.7 (1.4)	0.035

*Maternal baseline characteristics were measured during 11–13^+ 6^ weeks’ gestation. (SD: standard deviation; CRL: crown-rump length; NT: nuchal translucency )

**Fig. 2:**
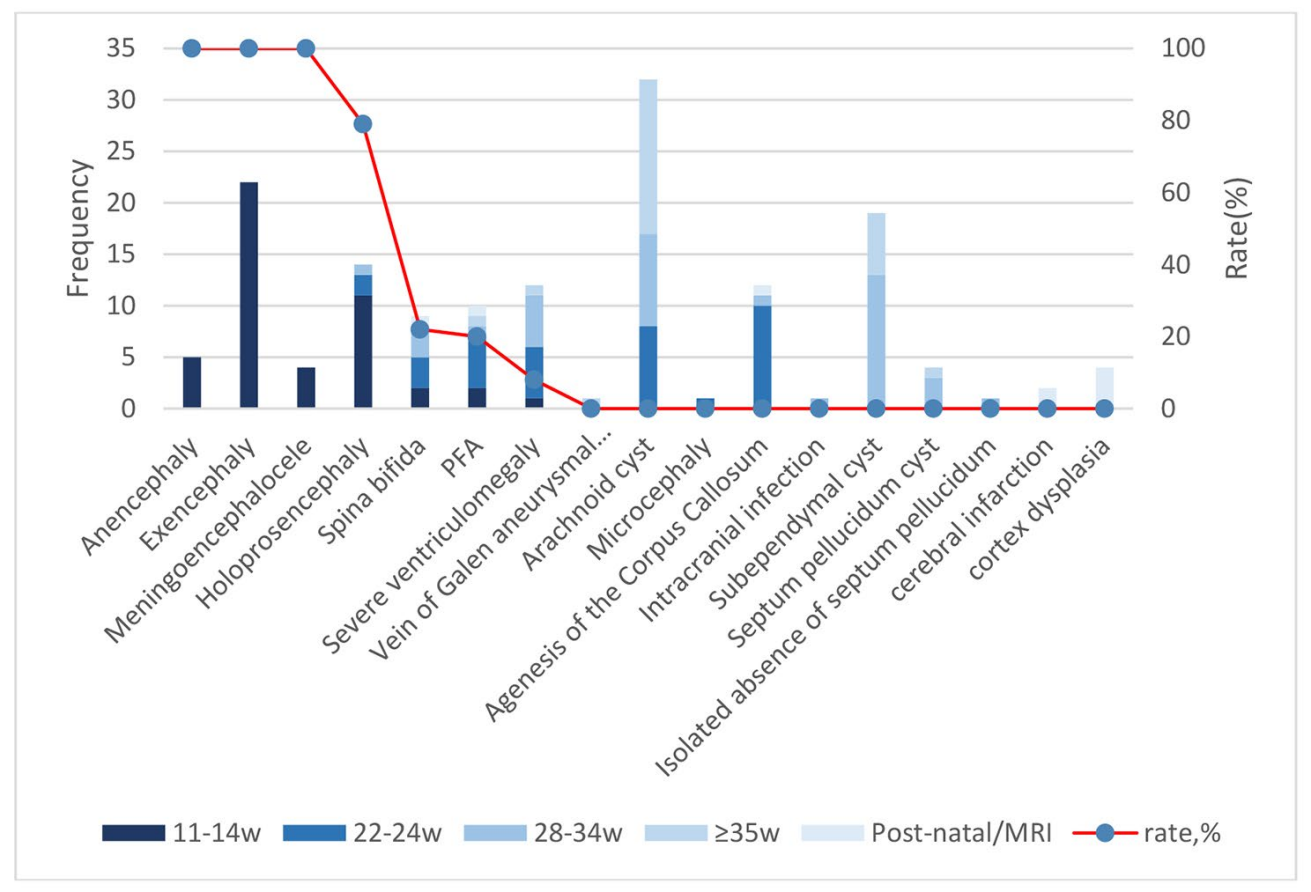
The cases of CNS abnormalities detected at each stage and the detection rates of different kinds of CNS anomalies during the first trimester PFA: posterior cranial fossa anomalies

The categories and their detection rate of CNS abnormalities diagnosed during the first-trimester scan are shown in Table 2. The detection of fetuses with CNS abnormalities at each stage and the diagnosis rate of different kinds of CNS anomalies during the first trimester are shown in Fig. 2. The detection rates of CNS anomalies during first trimester, second trimester, third trimester and late third trimester was 32%, 22%, 25% and 16%, respectively. And there were 5% of CNS anomalies missed by prenatal ultrasound.

**Table 2:** Fetal CNS abnormalities diagnosed during first-trimester scans

Fetal CNS abnormality	Total	Increased NT	Diagnosis						Pregnancy outcome		
			11-14w	rate, %	22-24w	28-34w	≥ 35w	Post-natal/MRI	TOP	Misc/IUD	LB
Anencephaly	5		5	100	0	0	0		5	0	0
Exencephaly	22	1	22	100	0	0	0		22	0	0
Meningoencephalocele	4	1	4	100	0	0	0		3	1	0
Holoprosencephaly	14		11	79	2	1	0		14	0	0
Alobar	5		5	100	0	0	0		5	0	0
Semi lobar	8		6	75	2	0	0		8	0	0
Lobar	1		0	0	0	1	0		1	0	0
Spina bifida	9		2	22	3	3	0	1	5	0	4
Open	3		2	67	1	0	0		3	0	0
Closed	6		0	0	2	3	0	1	2	0	4
PFA	10	1	2	20	5	1	1	1	5	0	5
Dandy-Walker	1		1	100	0	0	0		1	0	0
Vermis hypoplasia	1		0	0	1	0	0		1	0	0
Joubert Syndrome	1		1	100	0	0	0		1	0	0
Cerebellar hypoplasia	1		0	0	1	0	0		1	0	0
Blake’s cyst	5	1	0	0	3	1	1		1	0	4
Mega Cisterna Magna	1		0	0	0	0	0	1	0	0	1
Severe ventriculomegaly	12	1	1	8	5	5	1		10	1	1
Vein of Galen aneurysmal malformation	1		0	0	0	0	1		0	0	1
Arachnoid cyst	32	2	0	0	8	9	15		6	0	26
Microcephaly	1		0	0	1	0	0		1	0	0
Agenesis of the Corpus Callosum	12	2	0	0	10	1	0	1	10	0	2
Intracranial infection	1		0	0	0	1	0		0	0	1
Subependymal cyst	19		0	0	0	13	6		1	0	18
Septum pellucidum cyst	4		0	0	0	3	1		0	0	4
Isolated absence of septum pellucidum	1		0	0	0	1	0		0	0	1
cerebral infarction	2		0	0	0	0	0	2	1	1	0
cortex dysplasia	4		0	0	0	0	0	4	3	1	0

NT: nuchal translucency; PFA: posterior cranial fossa anomalies; TOP: Termination Of Pregnancy; Misc: Miscarriage; IUD: Intrauterine Death; LB: Live Birth

Exencephaly, anencephaly, meningoencephalocele and alobar holoprosencephaly were fully discovered at the first-trimester scan. Some kinds of CNS malformations could be partially detected at the first trimester scan, including posterior cranial fossa anomalies (PFA), open spina bifida, semi-lobar holoprosencephaly, and severe ventriculomegaly. Some CNS anomalies were never detected in the first trimester, such as vein of Galen aneurysmal malformation, closed spina bifida, lobar holoprosencephaly, intracranial infection, arachnoid cyst, agenesis of the corpus callosum, septum pellucidum cyst, isolated absence of septum pellucidum and so on. Some kinds of CNS anomalies could still be detected in the third trimester among the cases with no obvious abnormalities in the second-trimester scan, such as vein of Galen aneurysmal malformation, closed spina bifida, lobar holoprosencephaly, severe ventriculomegaly, intracranial infection, arachnoid cyst, agenesis of the corpus callosum and septum pellucidum cyst. The false positive rate for the diagnosis of CNS abnormalities by the first trimester scan in this study was 0% and the false negative rate was 68.1%.

**Fig. 3:**
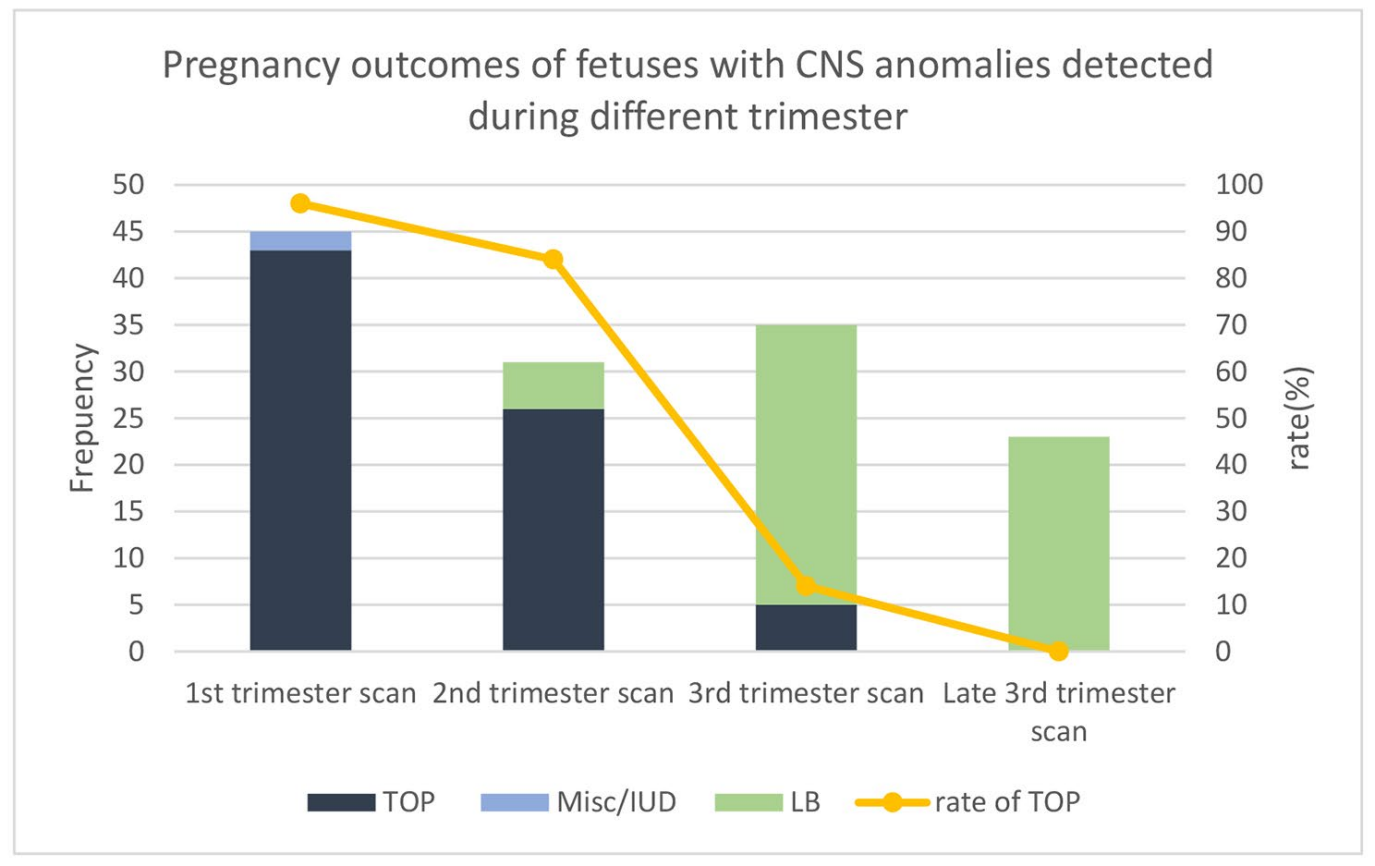
Pregnancy outcomes of fetuses with CNS anomalies detected at different trimesters and the rate of TOP TOP: Termination Of Pregnancy; Misc: Miscarriage; IUD: Intrauterine Death; LB: Live Birth

The pregnancy outcomes of the cases with CNS anomalies detected during the first, second, third, and late third trimester are shown in Table 3; Fig. 3.

**Table 3:** The pregnancy outcomes of the cases with CNS anomalies

Gestational age of diagnosed or suspected abnormality	Number of cases with CNS anomalies (detection rate)	Pregnancy outcome		
		TOP	Misc/IUD	LB
1st trimester scan	45(32%)	43(96%)	2(4%)	0
2nd trimester scan	31(22%)	26(84%)	0	5(16%)
3rd trimester scan	35(25%)	5(14%)	0	30(86%)
Late 3rd trimester scan	23(16%)	0	0	23(100%)

TOP: Termination Of Pregnancy; Misc: Miscarriage; IUD: Intrauterine Death; LB: Live Birth

Regarding invasive testing, 38 cases were missed, 52 were not performed, 42 had normal results and 9 had abnormal results, including 2 cases of trisomy 21, 1 case of trisomy 18, 1 case of chromosome balanced translocation, 2 cases of copy number variation and 2 cases with unknown details. 5 of the chromosomal abnormalities were positive on the first trimester ultrasound.

## Discussion

A 3-year experience from a tertiary center using an unselected cohort has shown that almost 1/3 of central nervous system anomalies were detected by the standard first-trimester scan. The diagnostic rate of CNS abnormalities in the first trimester in our study (32%) is slightly lower than in a large sample study from King’s College Hospital, London, UK (51.6%) [21]. This may be due to the different types of diseases included in the studies and the different incidence of neural tube defects which could influence the detection rate of first-trimester scan in particular. However, the rate of severe CNS malformations was similar, including anencephaly, exencephaly, alobar holoprosencephaly, open spina bifida (59.3%). We diagnosed all cases of anencephaly, exencephaly, alobar holoprosencephaly and meningoencephalocele, and some cases of open spina bifida, posterior cranial fossa anomalies, semi-lobar holoprosencephaly and severe ventriculomegaly. This is consistent with previous studies [17, 21, 22]. Therefore, it is recommended that some severe structural abnormalities should be screened in the first trimester.

It has been reported that early recognition of open spina bifida (OSB) and other major CNS anomalies were allowed in first trimester, such as ventriculomegaly, Dandy–Walker malformation, hypoplasia of the vermis or cerebellum and so on [17, 21, 23]. Observation of the brainstem, fourth ventricle and cisterna magna helped to increase the diagnostic rate of open spina bifida and posterior fossa malformations. [9, 24] Qualitative observation of the three spaces of posterior fossa detected 67% (2/3) of spina bifida and 20% (2/10) of posterior cranial fossa anomalies in this study. Two cases of posterior cranial fossa anomalies were suspected at first trimester scan in this study, and were confirmed as Dandy-Walker malformation and Joubert Syndrome respectively at the second trimester follow-up scan. However, it should be noted that false negatives or false positives may occur. Abnormalities have been suspected because some abnormal signs have been observed, but the fetal cerebellum and spine have not developed well in the first trimester. In these cases, a follow-up scan should be recommended. The false positive rate of this study was 0. This may be because only qualitative observation was made, with no quantitative judgement, only obvious structural changes can be detected. Or the sample size was insufficient. More research is needed.

Some CNS anomalies were never discovered during the first trimester due to incomplete fetal brain development and no obvious abnormal predictive signs during the first trimester. What’s more, some kinds of CNS anomalies were not detected by ultrasound throughout pregnancy but by MRI in this study, such as cerebral infarction and cortical dysplasia. However, in some of these cases, ultrasound revealed the abnormal middle cerebral artery blood flow or small biparietal diameter and head circumference. This demonstrated that MRI could be an adjunct, which were able to detect additional lesions that were either suspected or missed on US, including destructive lesions, cortical abnormalities, corpus callosum anomalies, and posterior fossa anomalies, as previous studies have shown [25–27]. Therefore, MRI should be recommended in fetuses suspected of having CNS abnormalities on ultrasonography.

Therefore, further improvements to the protocol should be explored to increase the detection rate of CNS anomalies and reduce false-negative rate. Quantitatively assessing intracranial structures may be useful, as Vayna’s study reported that assessing the BS to BSOB ratio, all cases of OSB were detected in the first trimester [17]. In addition, the new technique or valuable indicators probably help to advance the diagnosis of CNS anomalies in the first trimester, which could reduce the pain and anxiety of pregnant women. Hakan Kalaycı et al. found that the visualization of the pericallosal artery path (98% sensitivity) and calculation of the midbrain: falx ratio < 95th percentile (79–100% sensitivity), suggesting no elevation of the third ventricle and thalamus, had a very high sensitivity, indirectly confirming the presence of the corpus callosum in the first trimester of pregnancy [28]. P Martinez-Ten et al. have suggested that non-visualization of the choroid plexus of the fourth ventricle in the first trimester appears to be a strong marker for posterior fossa abnormalities and chromosomal defects [29]. And low torcular Herophili position and large brainstem-tentorium angle was reported to be the sign of open spinal dysraphism [13]. There are many more markers that we need to explore. The authors suggest that combined, quantitative assessment of multiple indicators may further improve the diagnostic power of ultrasound in early pregnancy. Unfortunately, these were not included in this study, which may be the direction of our future research efforts.

The termination of pregnancy (TOP) rate of fetal CNS anomalies detected by prenatal ultrasound in the first trimester, second trimester, and third trimester was 96%, 84% and 14%, respectively. The result showed that fetuses with CNS anomalies detected at first trimester scan were associated with a high rate of termination of pregnancy (96%) as an outcome. Fetuses with CNS anomalies which were detected at third trimester scan had a low probability of TOP (9%). While most of these were intracranial cystic disorders including arachnoid cyst, subependymal cyst, etc., the prognosis of fetuses with CNS anomalies detected at third trimester scan is usually good. Follow-up ultrasonography should be recommended in these cases. Fetal intracranial cystic disease may be transient, with some cysts resolving spontaneously with normal pediatric follow-up. This demonstrates that early screening for fetal abnormalities can provide reassurance to at-risk mothers who have previously had problematic pregnancies. This significantly minimizes unnecessary patient anxiety.

One of our strengths is that a large number of pregnancies were included in our study. And all continuing pregnancies underwent a series of prenatal ultrasound scans at 11–14, 20–24, 28–34 and 34–38 weeks of gestation by sonographers who had undergone rigorous training and passed the qualifying examination. What’s more, we describe the detection of more detailed categories of fetal CNS abnormalities and pregnancy outcomes detected in each trimester. In addition to its retrospective nature, this study has several limitations. First, there may be false negatives. Some kinds of CNS anomalies may be missed because these internal abnormalities were rarely examined in the absence of clinical symptoms. Second, some abnormal cases were confirmed by trained ultrasound specialists when both autopsy and MRI results could not be obtained.

## Conclusions

A 3-year experience from a tertiary center using an unselected cohort has shown that almost 1/3 of central nervous system anomalies were detected by the standard first-trimester scans. Some anomalies were easily detected, such as exencephaly, anencephaly, alobar holoprosencephaly and meningoencephalocele. Fetuses with CNS anomalies detected at first trimester scan were associated with a high rate of abortion. Early screening for fetal abnormalities gives parents more time for medical consultation and, if necessary, safer abortion. Therefore, it is recommended that some severe CNS anomalies should be screened in the first trimester. The standardized anatomical protocol, consisting of four fetal brain planes, was recommended for routine first-trimester ultrasound screening in our study.

## Data Availability

The datasets used and/or analyzed during the current study are available from the corresponding author on reasonable request.

## List of Abbreviations

NT: Nuchal translucency
CNS: Central nervous system
BS: Brain stem
BSOB: Brain stem-to-occipital bone
IT: Intracranial translucency
CM: Cisterna magna
CRL: Crown-Rump Length
MRI: Magnetic resonance imaging
SD: Standard deviation
TOP: Termination of pregnancy
PFA: Posterior cranial fossa anomalies.
